# Supplementary material for: Causal Relationship between Aging and Anorexia Nervosa: A White-Matter-Microstructure-Mediated Mendelian Randomization Analysis
Source: Biomedicines. 2024 Aug 16;12(8):1874. doi: 10.3390/biomedicines12081874 (PMC11351342; doi:10.3390/biomedicines12081874)
Supplement: Supplementary file 1 [file biomedicines-12-01874-s001.zip › Supplementary Figure/Figure S4/Singe SNP plot of MR analysis for TL to ubm-b-1943.pdf]

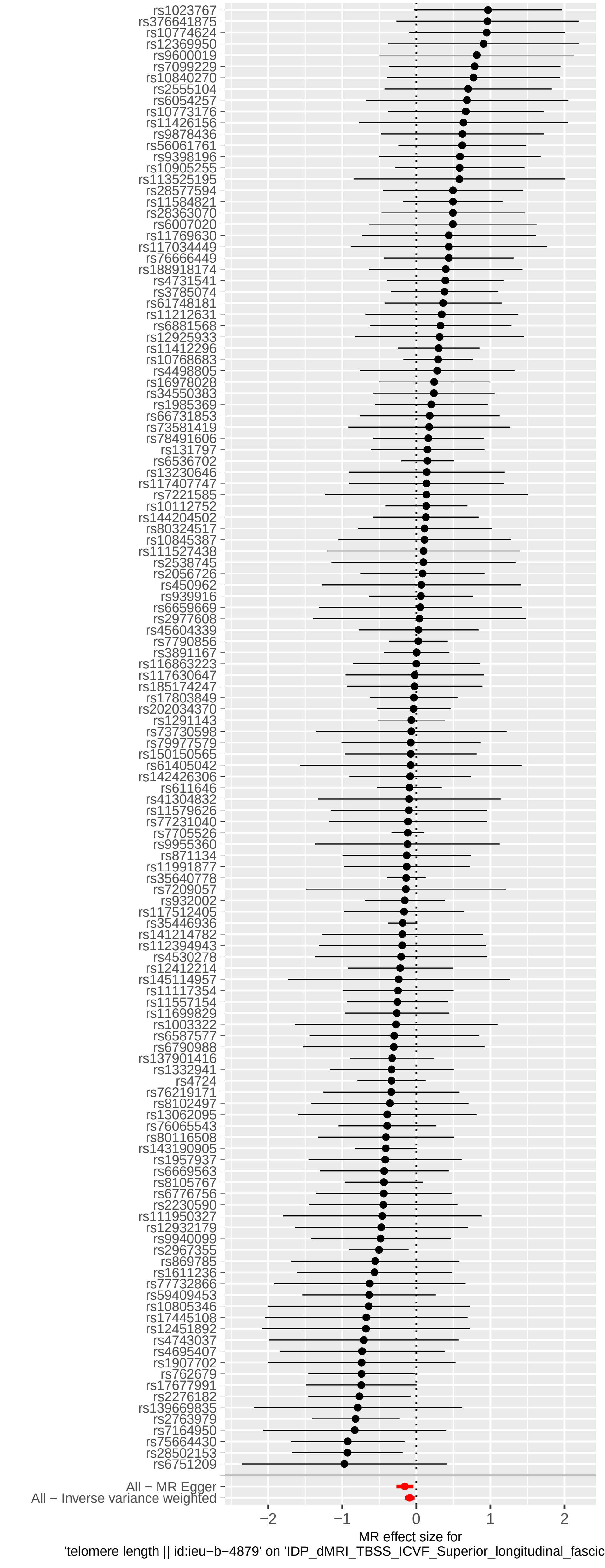
rs1023767
rs376641875
rs10774624
rs12369950
rs9600019
rs7099229
rs10840270
rs2555104
rs6054257
rs10773176
rs11426156
rs9878436
rs56061761
rs9398196
rs10905255
rs113525195
rs28577594
rs11584821
rs28363070
rs6007020
rs11769630
rs117034449
rs76666449
rs188918174
rs4731541
rs3785074
rs61748181
rs11212631
rs6881568
rs12925933
rs11412296
rs10768683
rs4498805
rs16978028
rs34550383
rs1985369
rs66731853
rs73581419
rs78491606
rs131797
rs6536702
rs13230646
rs117407747
rs7221585
rs10112752
rs144204502
rs80324517
rs10845387
rs111527438
rs2538745
rs2056726
rs450962
rs939916
rs6659669
rs2977608
rs45604339
rs7790856
rs3891167
rs116863223
rs117630647
rs185174247
rs17803849
rs202034370
rs1291143
rs73730598
rs79977579
rs150150565
rs61405042
rs142426306
rs611646
rs41304832
rs11579626
rs77231040
rs7705526
rs9955360
rs871134
rs11991877
rs35640778
rs7209057
rs932002
rs117512405
rs35446936
rs141214782
rs112394943
rs4530278
rs12412214
rs145114957
rs11117354
rs11557154
rs11699829
rs1003322
rs6587577
rs6790988
rs137901416
rs1332941
rs4724
rs76219171
rs8102497
rs13062095
rs76065543
rs80116508
rs143190905
rs1957937
rs6669563
rs8105767
rs6776756
rs2230590
rs111950327
rs12932179
rs9940099
rs2967355
rs869785
rs1611236
rs77732866
rs59409453
rs10805346
rs17445108
rs12451892
rs4743037
rs4695407
rs1907702
rs762679
rs17677991
rs2276182
rs139669835
rs2763979
rs7164950
rs75664430
rs28502153
rs6751209
All − MR Egger
All − Inverse variance weighted
−2
−1
0
1
2
MR effect size for
'telomere length || id:ieu−b−4879' on 'IDP_dMRI_TBSS_ICVF_Superior_longitudinal_fascic
